# Supplementary material for: From transistor to trapped-ion computers for quantum chemistry
Source: Sci Rep. 2014 Jan 7;4:3589. doi: 10.1038/srep03589 (PMC5378044; doi:10.1038/srep03589)
Supplement: Supplementary Information — Supplementary Material [file srep03589-s1.pdf]

From transistor to trapped-ion computers for quantum chemistry

M.-H. Yung,[1, 2, *] J. Casanova,[3, *] A. Mezzacapo,[3] J. McClean,[2] L. Lamata,[3] A. Aspuru-Guzik,[2, †] and E. Solano[3, 4, ‡]

[1] *Center for Quantum Information, Institute for Interdisciplinary Information Sciences, Tsinghua University, Beijing, 100084, P. R. China*

[2] *Department of Chemistry and Chemical Biology, Harvard University, Cambridge MA, 02138, USA*

[3] *Department of Physical Chemistry, University of the Basque Country UPV/EHU, Apartado 644, 48080 Bilbao, Spain*

[4] *IKERBASQUE, Basque Foundation for Science, Alameda Urquijo 36, 48011 Bilbao, Spain*

(Dated: December 1, 2013)

---

* These authors contributed equally to this work.

† aspuru@chemistry.harvard.edu

‡ enrique.solano@ehu.es

SUPPLEMENTARY MATERIAL

In this Supplementary Material we give further details of our proposal, including a thorough explanation of the quantum simulation of molecules involving fermionic and bosonic degrees of freedom with trapped ions, and electric dipole transition measurements with a trapped-ion quantum simulator.

A. Quantum simulation

In general, quantum simulation can be divided into two classes, namely analog and digital. Analog quantum simulation requires the engineering of the Hamiltonian of a certain system to mimic the Hamiltonian of a target system. Digital quantum simulation employs a quantum computer, which decomposes the simulation process into pieces of sub-modules such as quantum logic gates. However, the use of quantum logic gates is not absolutely necessary for digital quantum simulation. For example, consider the case of trapped ions; we will see that certain simulation steps requires us to apply quantum logic gates to implement fermionic degrees of freedom, together with some quantum operations for controlling the vibronic degrees of freedom, which are analog and will implement bosonic modes.

For simulating quantum chemistry, it is possible to work in either the first-quantization representation or the second-quantization representation. This work mainly includes the latter approach, because the number of qubits required is less than that in the former approach, especially when low-energy state properties are considered. However, we note that many techniques described here are also applicable for the first-quantization approach.

B. Computational complexity of quantum chemistry

To the best of our knowledge, there is no rigorous proof showing that quantum computers are capable of solving all ground-state problems in quantum chemistry. Instead, some results indicate that some ground-state problems in physics and chemistry are computationally hard problems [1]. For example, the N-representability problem is known to be QMA-complete, and finding the universal functional in density functional theory is known to be QMA-hard. In spite of the negative results, quantum computers can still be valuable for solving a wide range of quantum chemistry problems. These include ground state energy computations [2, 3], as well as molecular dynamics [4].

C. Simulating electronic structure involving molecular vibrations

After the potential surface is constructed by the electronic method, we can include the effect of molecular vibrations by local expansion, e.g. near the equilibrium position, as we show below.

1. *Electronic transitions coupled with nuclear motion*

We point out that within the Born-Oppenheimer approximation, the molecular vibronic states are of the form, $\phi_n(\mathbf{r},\mathbf{R})\,\chi_{n,v}(\mathbf{R})$ where $\mathbf{r}$ and $\mathbf{R}$ respectively refers to the electronic and nuclear coordinates. The eigenfunctions $\phi_n(\mathbf{r},\mathbf{R})$ of the electronic Hamiltonian are obtained at a fixed nuclear configuration. The nuclear wavefunction $\chi_{n,v}(\mathbf{R})$, for each electronic eigenstate n, is defined through a nuclear potential surface $E_{\mathrm{el}}^{(n)}(\mathbf{R})$, which is also one of the eigenenergies of the electronic Hamiltonian.

With a quantum computer, the potential energy surface that corresponds to different electronic eigenstates can be systematically probed using the phase estimation method. We can then locate those local minima where the gradient of the energy is zero, and approximate up to second order in $\delta R_\alpha \equiv R_\alpha - R_{\alpha *}$, the deviation of the nuclear coordinate R_α from the equilibrium configuration $R_{\alpha *}$. The energy surface can be modeled as

$$E_{\mathrm{el}}^{(n)}(\mathbf{R}) \approx E_{\mathrm{el}}^{(n)}(\mathbf{R}_*^{(n)}) + \sum_{\alpha,\beta} D_{\alpha\beta}(\mathbf{R}_*^{(n)})\delta R_\alpha \delta R_\beta\,, \tag{1}$$

where $D_{\alpha\beta}(\mathbf{R}_*^{(n)}) \equiv (1/2)\,\partial^2 E_{el}^{(n)}(\mathbf{R}{=}\mathbf{R}_*^{(n)})/\partial R_\alpha \partial R_\beta$ is the Hessian matrix. With a change of coordinates for the Hessian matrices, we can always choose to work with the normal modes $\mathbf{x}^{(n)} = \{x_\alpha^{(n)}\}$ for each potential energy

surface, such that

$$E_{\mathrm{el}}^{(n)}(\mathbf{x}^{(n)}) \approx E_{\mathrm{el}}^{(n)}(\mathbf{R}_*^{(n)}) + \frac{1}{2}\sum_\alpha m_\alpha \omega_\alpha^{(n)2} x_\alpha^{(n)2}. \tag{2}$$

Most of the important features of vibronic coupling can be captured by considering the transition between two Born-Oppenheimer electronic levels [5]. In the following, we will focus on the method of simulation of the transition between two electronic levels, labeled as $|\uparrow\rangle$ and $|\downarrow\rangle$, when perturbed by an external laser field. The Hamiltonian of the system can be written as

$$H = |\downarrow\rangle\langle\downarrow| \otimes H_G + |\uparrow\rangle\langle\uparrow| \otimes H_E, \tag{3}$$

where $H_G \equiv \Delta_g + H_g$ is the Hamiltonian for the nuclear motion in the electronic ground state and similarly $H_E \equiv \Delta_e + H_e$ is the nuclear Hamiltonian in the excited state. Here Δ_g and Δ_e are the energies of the two bare electronic states. In the second-quantized representation,

$$H_g = \sum_k \omega_k^{(g)} a_k^\dagger a_k \quad \text{and} \quad H_e = \sum_k \omega_k^{(e)} b_k^\dagger b_k \tag{4}$$

are diagonal, as viewed from their own coordinate systems. However, in general, the two sets of normal modes are related by rotation and translation, which means that a transformation of the kind $b_k = \sum_j s_{kj} a_j + \lambda_k$ is needed for unifying the representations (see Secs. E and F in this Supplementary Material).

To illustrate our method of quantum simulation with trapped ions, it is sufficient to consider one normal mode (for example, linear molecules). For this case, we assume $H_g = \omega^{(g)} a^\dagger a$, $H_e = \omega^{(e)} b^\dagger b$, and $b = a + \lambda$ where λ is a real constant. From Eq. (3), we need to simulate the following Hamiltonian,

$$H = H_S + \Omega(\sigma_z)\, a^\dagger a + \tfrac{1}{2}\lambda\omega^{(e)} (I + \sigma_z)\left(a^\dagger + a\right), \tag{5}$$

where the term $H_S = \frac{1}{2}(\Delta_g - \Delta_e)\sigma_z$ contains only local terms of the spin, and $\Omega(\sigma_z) = \frac{1}{2}\left(\omega^{(g)} + \omega^{(e)}\right) I + \frac{1}{2}\left(\omega^{(g)} - \omega^{(e)}\right)\sigma_z$ represents a spin-dependent frequency for the effective boson mode.

In order to examine the response of the system under external pertubations, we consider the dipole correlation function

$$C_{\mu\mu}(t) = \sum_n p_n \langle n, \downarrow| e^{iHt} \mu e^{-iHt} \mu |n, \downarrow\rangle . \tag{6}$$

Under the Condon approximation, assuming real electronic eigenstates, the dipole operator μ has the form,

$$\mu = \mu_{ge}\left(|\downarrow\rangle\langle\uparrow| + |\uparrow\rangle\langle\downarrow|\right) = \mu_{ge}\sigma_x. \tag{7}$$

Thus, the problem of simulating absorption resulting from the coupling of electronic and nuclear motion in chemistry reduces to computing expectation values of the unitary operator

$$U_d = e^{iHt}\sigma_x e^{-iHt}\sigma_x, \tag{8}$$

and weighting the final result by $p_n\mu_{ge}^2$. The final spectrum is, of course, obtained through a Fourier transform

$$\sigma_{abs}(\omega) = \int_{-\infty}^{\infty} dt\; e^{-i\omega t} C_{\mu\mu}(t). \tag{9}$$

2. *Simulation of vibronic coupling with trapped ions*

The dynamics associated with the Hamiltonian in Eq. (5) can be generated easily with two trapped ions. As H_S commutes with the rest of the terms in Eq. (5), it can be eliminated via a change to an interaction picture. Considering a digital quantum simulation protocol, the remaining task is to implement the interactions $\exp[-i\Omega(\sigma_z)\, t a^\dagger a]$ and $\exp[-i\lambda\omega^{(e)}(I + \sigma_z)\left(a^\dagger + a\right) t/2]$ in trapped ions. The first one corresponds to the evolution associated with a detuned red sideband excitation applied to one of the ions (a dispersive Jaynes-Cummings interaction), and a rotation of its internal state in order to eliminate the residual projective term. To implement the second term we will use both ions.

The term related to the operator $\sigma_z(a^\dagger + a)$ corresponds to the evolution under red and blue sideband excitations applied to one of the ions (a Jaynes-Cummings and anti Jaynes-Cummings interactions with appropriate phases). We will use the second ion to implement the term $(a^\dagger + a)$. The latter can be generated by applying again the same scheme of lasers that generates the interaction $\sigma_z(a^\dagger + a)$ where now the operator σ_z acts on the internal state of the second ion. Preparing this state in an eigenstate of σ_z one obtains the desired effective Hamiltonian. As we have shown here, one of the main appeals of a quantum simulation of quantum chemistry with trapped ions is the possibility to include fermionic (electronic) as well as bosonic (vibronic) degrees of freedom, in a new kind of mixed digital-analog quantum simulator. The availability of the motional degrees of freedom in trapped ions, that straightforwardly provide the bosonic modes in an analog way, makes this system especially suited for simulating this kind of chemical problems.

D. Electric transition dipoles through weak measurement

Here we sketch the method for obtaining the transition dipole between a pair of electronic states $|g\rangle$ and $|e\rangle$. This method is similar, although not identical, to the weak measurement method using a qubit as a measurement probe. To make the presentation of our method more general, our goal is to measure the matrix element $\langle e| A |g\rangle$ for any given Hermitian matrix A. We assume that a potential energy surface between these two electronic levels is probably scanned, and the energy levels for higher excited states can be ignored. Suppose we started with a reasonable good approximation of the ground state $|g\rangle$, and we can prepare the exact ground state using the phase estimation algorithm. Then, we apply a weak perturbation λ, e.g. $e^{-i\lambda Q}$, to the ground state and obtain (to order $O(\lambda)$) the state $|i\rangle \equiv e^{-i\lambda Q} |g\rangle \approx |g\rangle + q\lambda |e\rangle$. Here λ is a small positive real number. The actual form of the Hermitian operator Q is not important, as long as $\langle e| Q |g\rangle \equiv iq \neq 0$. Note that the eigenstates are defined up a phase factor. Therefore, without loss of generality, we can assume q is a positive real number as well. In fact, the absolute value $|q|$ can be measured with repeated applications of the phase estimation algorithm.

Now, we prepare an ancilla qubit in the state $|+\rangle \equiv (|0\rangle + |1\rangle)/\sqrt{2}$, and apply a control-$U_A$, where $U_A \equiv e^{-i\lambda A}$. The resulting state becomes $(|0\rangle |i\rangle + |1\rangle U_A |i\rangle)/\sqrt{2}$. The phase estimation algorithm allows us to perform post-selection to project the system state to $|e\rangle$. The resulting state of the ancilla qubit is $\propto \langle e|i\rangle|0\rangle + \langle e|U_A|i\rangle|1\rangle$. To the first-order expansion in λ, we have (before normalization)

$$q\lambda |0\rangle + (q - i \langle e| A |g\rangle) \lambda |1\rangle , \tag{10}$$

where we used $\langle e|i\rangle = q\lambda$, and $\langle e| U_A |i\rangle = \langle e| U_A |g\rangle + q\lambda \langle e| U_A |e\rangle = -i \langle e| A |g\rangle \lambda + q\lambda$. Since the value of q is known, a state tomography on the ancilla qubit state reveals the value of the matrix element $\langle e| A |g\rangle$.

Returning to the case of the electric dipole moment, it is defined as $\mu \equiv -e \sum_i \mathbf{r}_i$. In the second quantized form is $\mu = \sum_{pq} u_{pq} a_p^\dagger a_q$, where $u_{pq} \equiv -e \int \phi_p^* (\mathbf{r}) \mathbf{r} \phi_q (\mathbf{r})\, d\mathbf{r}$ is nothing but the single-particle integral. The simulation of the corresponding operator $U_A \equiv e^{-i\lambda A}$, with A replaced by μ, can be performed efficiently after performing the Jordan-Wigner transformation.

E. Derivation of the spin-boson coupling

Consider the full Hamiltonian of two potential energy surfaces,

$$H = |\downarrow\rangle \langle\downarrow| \otimes H_G + |\uparrow\rangle \langle\uparrow| \otimes H_E, \tag{11}$$

where

$$H_G \equiv \Delta_g + H_g \tag{12}$$

is the Hamiltonian for the nuclear motion in the electronic ground state and similarly

$$H_E \equiv \Delta_e + H_e \tag{13}$$

is the nuclear Hamiltonian in the excited state. Here Δ_g and Δ_e are the zero-point energies of the two potential energy surfaces. In the second-quantized representation, we consider one normal mode for each local minimum in the potential energy surface,

$$H_g = \omega^{(g)} a^\dagger a \quad \text{and} \quad H_e = \omega^{(e)} b^\dagger b. \tag{14}$$

Here the two normal modes are related by a shift of a real constant λ, namely

$$b = a + \lambda. \tag{15}$$

Now, we will rewrite the full Hamiltonian in terms of the Pauli matrix

$$\sigma_z = \begin{pmatrix} 1 & 0 \\ 0 & -1 \end{pmatrix} = \left|\uparrow\right\rangle\left\langle\uparrow\right| - \left|\downarrow\right\rangle\left\langle\downarrow\right|. \tag{16}$$

First of all, we write $H = H_{SB} + H_S$, where

$$H_{SB} = \left|\downarrow\right\rangle\left\langle\downarrow\right| \otimes \omega^{(g)} a^\dagger a + \left|\uparrow\right\rangle\left\langle\uparrow\right| \otimes \omega^{(e)} b^\dagger b, \tag{17}$$

and

$$\begin{aligned} H_S &\equiv \left|\downarrow\right\rangle\left\langle\downarrow\right|\Delta_g + \left|\uparrow\right\rangle\left\langle\uparrow\right|\Delta_e \\ &= \frac{1}{2}\left(\Delta_g + \Delta_e\right) I + \frac{1}{2}\left(\Delta_g - \Delta_e\right)\sigma_z. \end{aligned} \tag{18}$$

Next, we use Eq. (15) to write H_{SB} as

$$H_{SB} = \Omega\left(\sigma_z\right) \otimes a^\dagger a + \tfrac{1}{2}\lambda\omega^{(e)}\left(I + \sigma_z\right) \otimes \left(a^\dagger + a\right), \tag{19}$$

where the frequency of the effective mode becomes spin-dependent,

$$\begin{aligned} \Omega\left(\sigma_z\right) &\equiv \left|\uparrow\right\rangle\left\langle\uparrow\right|\omega^{(g)} + \left|\downarrow\right\rangle\left\langle\downarrow\right|\omega^{(e)} \\ &= \frac{1}{2}\left(\omega^{(g)} + \omega^{(e)}\right) I + \frac{1}{2}\left(\omega^{(g)} - \omega^{(e)}\right)\sigma_z. \end{aligned} \tag{20}$$

F. Multimode extension of simulating vibronic coupling

In order to extend the method of simulating vibronic coupling to the case with multiple bosonic modes, we now consider the case of Eq. 4. If we express the excited state modes in terms of the ground state modes such that

$$b_k = \sum_j s_{kj} a_j + \lambda_k, \tag{21}$$

we can write H as

$$H = H_s' + \left|\downarrow\right\rangle\left\langle\downarrow\right| \otimes H_G + \left|\uparrow\right\rangle\left\langle\uparrow\right| \otimes H_E, \tag{22}$$

where

$$H_G \equiv \sum_k \omega_k^{(g)} a_k^\dagger a_k, \tag{23}$$

and

$$H_E \equiv \sum_{kjl} \omega_k^{(e)} s_{kj} s_{lk} a_j^\dagger a_l + \sum_{kj} \omega_k^{(e)} s_{kj}\lambda_k \left(a_j^\dagger + a_j\right). \tag{24}$$

In the definition of H_s', the only change from H_s is given by

$$\Delta_e' = \Delta_e + \sum_k \lambda_k^2. \tag{25}$$

With knowledge of s_{ij} and λ_i for all modes, we can then repeat the above procedure to determine the absorption spectrum for a complicated system using a quantum computer. The above Hamiltonian can be written in a form more familiar to quantum computation as

$$\begin{aligned} H = H_s' &+ \sum_k \Omega_k(\sigma_z) a_k^\dagger a_k \\ &+ \frac{1}{2}\sum_{kj} s_{kj}\omega_k^{(e)}\lambda_k (I + \sigma_z)(a_j^\dagger + a_j) \\ &+ \frac{1}{2}\sum_k \sum_{j \neq l} s_{kj} s_{lk}\omega_k^{(e)} (I + \sigma_z) a_j^\dagger a_l \end{aligned} \tag{26}$$

where we define

$$\Omega_k(\sigma_z) \equiv \frac{1}{2}(\omega_k^{(g)} + s_{kk}^2\omega_k^{(e)})I + \frac{1}{2}(\omega_k^{(g)} - s_{kk}^2\omega_k^{(e)})\sigma_z. \tag{27}$$

In cases where Duchinsky rotations of the normal modes can be neglected ($s_{ij} = \delta_{ij}$), this expression can be further reduced to

$$\begin{aligned} H = H_s' &+ \sum_k \Omega_k'(\sigma_z) a_k^\dagger a_k \\ &+ \frac{1}{2}\sum_k \omega_k^{(e)} \lambda_k (I + \sigma_z)(a_k^\dagger + a_k) \end{aligned} \tag{28}$$

with the simplification

$$\Omega_k'(\sigma_z) = \frac{1}{2}(\omega_k^{(g)} + \omega_k^{(e)})I + \frac{1}{2}(\omega_k^{(g)} - \omega_k^{(e)})\sigma_z. \tag{29}$$

---

[1] Whitfield, J.-D., Love, P. J., & Aspuru-Guzik, A. Computational Complexity in Electronic Structure. eprint arXiv:1208.3334 (2012).

[2] Abrams, D. & Lloyd, S. Quantum Algorithm Providing Exponential Speed Increase for Finding Eigenvalues and Eigenvectors. *Phys. Rev. Lett.* **83**, 5162–5165, (1999).

[3] Aspuru-Guzik, A., Dutoi, A. D., Love, P. J., & Head-Gordon, M. Simulated quantum computation of molecular energies. *Science* **309**, 1704–7 (2005).

[4] Love, P. J. Back to the Future: A roadmap for quantum simulation from vintage quantum chemistry. eprint arXiv:1208.5524 (2012). (To Appear in Advances in Chemical Physics)

[5] May, V. & Kühn, O. *Charge and Energy Transfer Dynamics in Molecular Systems.* (John Wiley & Sons, 2011).
